# Outcomes of Total Ankle Arthroplasty in Non-traumatic Arthritis Versus Post-traumatic Arthritis: A Systematic Review and Meta-Analysis

**DOI:** 10.7759/cureus.91680

**Published:** 2025-09-05

**Authors:** Tarik Al-Dahan, Sebastian Ndlovu, Baljinder Dhinsa, Crispin Southgate, Darren Geoffrey, Chiranjit De, Sathya Lakpriya, Nabil Seoudi, Rusul Al Bairmani

**Affiliations:** 1 Trauma and Orthopaedics, East Kent Hospitals University National Health Service (NHS) Foundation Trust, Ashford, GBR; 2 Emergency Medicine, University Hospital Southampton National Health Service (NHS) Foundation Trust, Southampton, GBR

**Keywords:** ankle arthritis, outcomes, post traumatic arthritis, total ankle arthroplasty, total ankle replacement

## Abstract

The objective of this review is to compare the outcomes of total ankle arthroplasty in patients with ankle arthritis without previous ankle trauma or intervention versus patients with post-traumatic arthritis. Further analysis was performed to review the outcomes of ankle arthroplasty irrespective of the patients’ cohorts.

A comprehensive literature search of Embase, Ovid Medline, Cumulative Index to Nursing and Allied Health Literature (CINAHL), Ovid Emcare and Cochrane, Turning Research Into Practice (TRIP) databases. In total, 17 studies met the inclusion criteria. Continuous data were pooled using the mean difference with a 95% Confidence interval. Dichotomous data were pooled as a risk ratio with a 95% Confidence interval, both using the Inverse variance random effect model.

The pooled analysis indicates a revision rate of 4.2% (14 of 330 patients) in the ankle arthritis without previous trauma or intervention group (POA) compared to 11.8% (29 of 245 patients) in the post-traumatic arthritis group (PTOA). There was no statistical difference in the American Orthopaedic Foot and Ankle Society (AOFAS) score between POA and PTOA groups (MD=1.0, 95% CI: 4.4 to 6.4) and no statistically significant difference in the complication rates between POA and PTOA (RR=0.79, 95% CI: 0.59 to 1.07).

This meta-analysis demonstrates that total ankle arthroplasty in patients with prior surgical intervention for post-traumatic arthritis is associated with higher revision rates than in patients without previous intervention. However, they might still yield a similar functional outcome in the short term. The role of total ankle arthroplasty should be clearly defined, weighing risks against patient expectations, with longitudinal studies needed to further clarify such risks and benefits.

## Introduction and background

Ankle osteoarthritis (OA) is a degenerative disease that generally affects older adults and results in the progressive loss of articular cartilage. Ankle OA is associated with significant pain and impact on quality of life, with physical and mental impacts estimated to be comparable to those of hip arthrosis [1]. In contrast to the hip, the incidence of primary osteoarthritis of the ankle is relatively rare, estimated at about 7% of all cases of end-stage ankle arthritis, with post-traumatic arthritis accounting for the majority of cases (70%-80%). Other secondary causes of ankle joint degeneration include dysplasia, inflammatory conditions, infection, haemophilia and vascular or neurological insults [2,3].

Treatment for ankle arthritis ranges from non-operative measures to operative options, which can include arthroscopic or open joint debridement, distraction arthroplasty, supramalleolar osteotomy, tibiotalar arthrodesis, structural tibiotalar allograft arthroplasty and total ankle arthroplasty. Ankle arthrodesis has reported good functional outcomes [4] and is considered by many as the gold standard option for young and active individuals. However, long-term follow-up studies have confirmed that arthrodesis can alter the biomechanics of the adjacent joints in the foot, such as the subtalar and talonavicular joints [5]. Whereas total ankle arthroplasty (TAA) is considered a viable alternative treatment option in patients with low functional requirements, minimal deformity and degenerative changes in other joints such as the ipsilateral hindfoot or midfoot (may require adjuvant procedures), or even contralateral ankle joint [6]. Long-term outcomes following TAA are still limited due to the lack of long-term quality evidence; however, studies have reported good short-term outcomes in terms of pain reduction, patient satisfaction and measured range of motion [6,7].

Although primary or idiopathic ankle osteoarthritis is considered to be rare, it can be argued that such cases may have unrecognised prior traumatic causal events. Regardless, supposed idiopathic ankle arthrosis is typically characterised by preserved anatomical alignment, and no significant fibrosis around the ankle joint, making surgical access easier through standard techniques [8]. In contrast, post-traumatic ankle arthritis (PTOA) typically occurs following significant fractures requiring surgical interventions. Patients who have PTOA often have compromised soft tissue envelopes due to scarring and multiple surgeries. Additionally, arthroplasty surgeons may face challenges in this cohort of patients such as bone deformities, loss of bone stock, and ligamentous damage which can significantly impact implant fixation and long-term outcomes after TAA [9].

However, there has been an evolution in ankle prosthesis design, with custom-alignment jigs, revision platforms and utilisation of talar replacements. First generation ankle prosthesis led to poor results [10]. As implant design and surgeons experience has improved, there has been a shift to accept ankle arthroplasty as an alternative to ankle arthrodesis in selected patients [11]. Predictably, there is clearly a paucity of evidence discussing the outcomes of TAA following ankle injuries requiring surgical intervention. The aim of this systematic review was to compare functional outcomes between patients’ ankle arthritis without previous trauma or intervention (POA) and PTOA to understand how the underlying pathophysiology affects the outcomes and complications. Ultimately, we aimed to indirectly determine whether TAA should be considered a safe and effective alternative to ankle arthrodesis in patients with PTOA who may or may not have undergone surgical intervention for their initial injury.

## Review

Methods

Search Strategy

A comprehensive literature search of Embase, Ovid Medline, Cumulated Index in Nursing and Allied Health Literature (CINAHL), Ovid Emcare and Cochrane, and Turning Research Into Practice (TRIP) databases was performed from inception until March 20, 2025, using the following search terms “Total Ankle Replacement”, “Total Ankle Arthroplasty” “Osteoarthritis”, “Post traumatic”. The detailed search query used for each database was illustrated in Supplementary Appendix. All duplicates of literature search results were removed, resulting in a final 112 references to review.

Screening was performed by two reviewers (T.A. and S.N.) in two steps. Initially, titles and abstract screening were performed, and then full-text screening for the eligible studies. A third senior reviewer (B.D.) was consulted when discrepancies arose between the two reviewers. This systematic review was conducted in accordance with the Preferred Reporting Items for Systematic Reviews and Meta-Analyses (PRISMA) [12]. We registered the review protocol with PROSPERO, the international registry for systematic review protocols (CRD420251020138).

**Figure 1:**
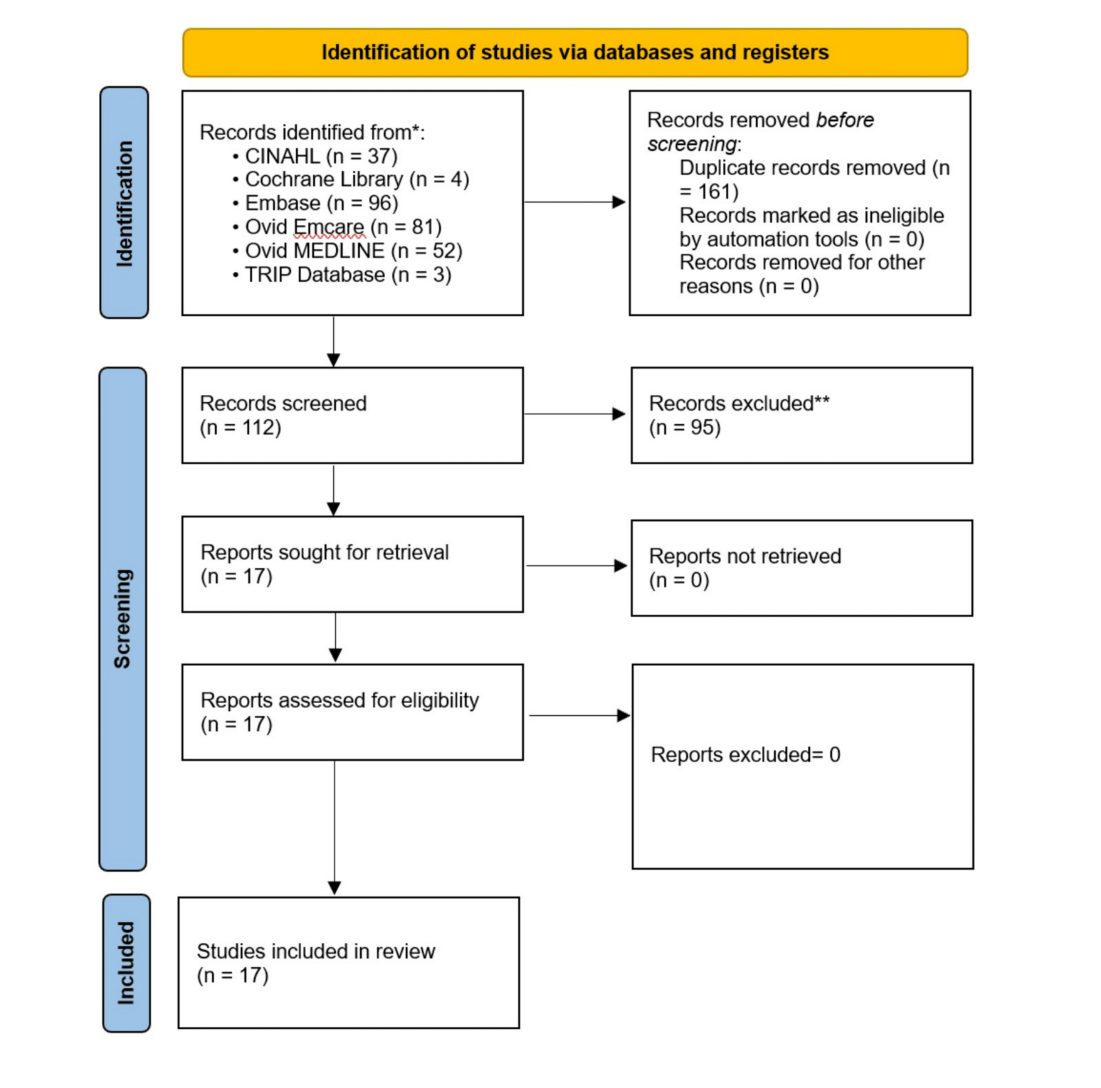
PRISMA flow diagram. PRISMA: Preferred Reporting Items for Systematic Reviews and Meta-Analyses. *Counts per database before deduplication. **Excluded at title/abstract screening.

Study Selection and Outcomes

All studies whose population underwent TAA for either arthritis without prior intervention (POA) or PTOA were included. When the studies included were single-arm studies comparing total ankle arthroplasty to another treatment option or lacked comparator, we extracted the data referring to the ankle replacement and excluded the other treatments to maximise data use and have broader understanding of the outcomes. The included double-arm studies compared outcomes of TAA in POA and PTOA groups. Studies with a minimum post-operative follow-up period of 36 months were included in the final analysis. In the single-arm analysis - to enhance generalisability of findings- we excluded studies that focused on specific implant type as they might reflect implant specific characteristics and techniques as we aimed to provide a more comprehensive assessment of the outcomes of the total ankle arthroplasty. Other exclusion criteria were conference abstracts, and studies not published in the English language or without an available English translation.

The primary outcomes were the complication rates, revision rate and a functional assessment tool such as the American Orthopaedic Foot and Ankle Society (AOFAS) Ankle-Hindfoot Score. As this score provides a clinically meaningful assessment to the pain, function and alignment of the hindfoot and ankle joint related to outcomes of ankle arthroplasty. and secondary outcome measures included reported complication rates, revision rate, malleolar fracture, Ankle Osteoarthritis Scale (AOS) score, Visual Analogue Scale (VAS) score and recorded range of movement (ROM) in terms of dorsiflexion and plantarflexion. All outcomes of interest were defined according to the study authors’ definitions. *Data Extraction and Quality Assessment*

Data extraction from included studies was performed in duplicate by two authors (D.G. and C.D.) using a specified online data extraction sheet. The extracted data included: (1) the characteristics of the included studies; (2) baseline characteristics of the included studies’ population; (3) risk of bias domains; (4) primary and secondary outcome measures. Quality of included studies was assessed either by the Newcastle-Ottawa Scale (NOS) for two-, three- and five-arm cohort studies, and National Institutes of Health (NIH) Quality Assessment Tool for single-arm cohort studies [13, 14] as shown in Tables 1, 2.

**Table 1:** Risk of bias of observational studies using the Newcastle Ottawa Scale (NOS) Quality assessed using the Newcastle-Ottawa Scale (NOS); maximum 9★ (Selection 4, Comparability 2, Outcome 3). Higher ★ = Lower risk of bias and higher methodological quality.

Study ID	Selection domain	Comparability domain	Outcome domain	Total Score	Overall Quality
Albagli et al. (2021) [15] (double-arm)	★★★★	★	★★	7	Good quality
Coetzee et al. (2020) [16] (three-arm)	★★★★	★	★	6	Fair quality
Johnson-Lynn et al. (2018) [17] (three-arm)	★★★★	★	★	6	Fair quality
Megerian et al. (2023) [18] (double-arm)	★★★	★★	★★	7	Good quality
Ramaskandhan et al. (2014) [19] (three-arm)	★★★★	★	★	6	Fair quality
Lee et al. (2019) [20] (double-arm)	★★★★	★★	★★	8	Good quality
Bai et al. (2010) [21] (double-arm)	★★★★	★	★★	7	Good quality
Bennett et al. (2017) [9] (five-arm)	★★★	★	★★	6	Good quality

**Table 2:** Risk of bias of observational studies using the National Institutes of Health (NIH) Quality Assessment Tool

Study	Clear Research Question?	Population Specified?	Participation ≥50%?	Same Population/Time?	Uniform Criteria?	Sample Size Justified?	Exposure Before Outcome?	Sufficient Timeframe?	Examined Exposure Levels?	Exposure Defined?	Exposure Assessed >1?	Outcomes Defined?	Assessors Blinded?	Loss ≤20%?	Adjusted Confounders?	Overall quality
Abarquero-Diezhandino et al. (2023) [22] (Single-arm)	Yes	Yes	Yes	Yes	Yes	No	Yes	Yes	No	Yes	No	Yes	No	Yes	No	Fair
Bardelli and Scoccianti (2006) [23] (Single-arm)	Yes	No	No	Yes	Unclear	No	Yes	Unclear	No	Partially	No	Partially	No	No	No	Poor
Brodsky et al. (2021) [24] (Single-arm)	Yes	Yes	Yes	Yes	Yes	No	Yes	Yes	No	Yes	No	Yes	No	Yes	No	Good
Lee et al. (2013)[25] (Single-arm)	Yes	Yes	Unclear	Yes	Yes	No	Yes	Yes	No	Partially	No	Yes	No	Unclear	No	Fair
Fischer et al. (2022) [26] (Single-arm)	Yes	Yes	Yes	Yes	No	No	Yes	Yes	Yes	Yes	No	Yes	No	Yes	No	Fair
Noelle et al. (2013) [27] (Single-arm)	Yes	Yes	No	Yes	Yes	No	Yes	Partial	NA	Yes	No	Yes	No	No	No	Fair
Rodrigues-Pinto et al. (2013) [28] (Single-arm)	Yes	Yes	No	Yes	Yes	No	Yes	Partial	NA	Yes	No	Yes	No	No	No	Good
Ross et al. (2021)[29] (Single-arm)	Yes	Yes	NA	No	No	No	Yes	Yes	NA	Yes	No	Partial	No	NA	Yes	Poor
Rybalko et al. (2018) [30] (Single-arm)	Yes	Partial	NA	No	No	No	Yes	No	NA	Partial	No	Yes	No	NA	No	Fair

Statistical Analysis

Statistical analysis was performed using Stata MP Version 18 for Windows (StataCorp, College Station, TX, USA). Continuous data was analysed using the mean and standard deviation (SD) changes from baseline, along with the total number of patients in each group, to calculate the standard mean difference (MD) between groups and its 95% confidence interval (95% CI) using the inverse variance method with a random-effects model. Dichotomous data were evaluated by pooling event frequencies and the total number of patients in each group to compute the risk ratio (RR) between groups and its 95% CI using the inverse variance method with a random-effects model.

Assessment of Heterogeneity

We used the chi-square (Cochrane Q) and I^2^ tests to assess statistical heterogeneity among our included studies. Significant heterogeneity was detected if the P-value of chi-square <0.1, and high heterogeneity was defined as I^2^ values of ≥50%.

Patient Characteristics

The initial search of databases revealed 112 records; however, only 17 studies, including 2,990 patients, met our inclusion criteria. Of these, nine were single-arm retrospective cohort studies [22-30], four were two-arm retrospective cohort studies [15,18,20,21], three were three-arm retrospective cohort studies [16,17,19], and one was a five-arm retrospective cohort study [9]. The PRISMA flow diagram for study selection is shown in Figure 1. Five studies use Salto Talaris prosthesis for total ankle replacement [16,24-26,28]. Three studies use MOBILITY Total Ankle System [9,17], and rest of the studies use other prostheses, including Scandinavian, Agility, PROPHECY INFINITY, and HINTEGRA prostheses. The mean follow-up period for all included studies was 39.48 months. The summary of the included studies is presented in Tables 3, 5. Of the 2,990 patients included in this systematic review, 32.85% were men and 67.15% were women, with average age from 18 to 94.5 years. Detailed baseline characteristics are shown in Tables 4, 6.

**Table 3:** Studies comparing the outcomes of total ankle arthroplasty (TAA) in non-traumatic osteoarthritis and post-traumatic osteoarthritis OA: Osteoarthritis; DJD: degenerative joint disease; PROM: Patient-Reported Outcome Measures.

Study ID	Study Design	Name of registry or study	Number of centres	Study time (recruitment time)	Total ankle replacement prosthesis	Inclusion criteria	Main findings (conclusion)	
								Megerian et al. (2023) [18] (Double-arm)	Retrospective cohort	NA	Two-canters	January 2014 to May 2022	Scandinavian Total Ankle Replacement (STAR)	All primary TAA patients conducted at two large hospital systems (one tertiary care academic medical centre and one county hospital).	Compared with POA, Fracture PTOA had higher complication and failure rates.	
Albagli et al. (2021) [15] (Double-arm)	Retrospective cohort	NA	Single centre	July 2015 to December 2017	PROPHECY INFINITY prosthesis	Patients underwent TAA using the PROPHECY INFINITY implant system that utilizes CT-based patient specific cutting guides.	Post-traumatic group shows satisfactory results in terms of patient satisfaction, short-term clinical and radiographic outcomes comparable to nontraumatic patients.	
Coetzee et al. (2020) [16] (Three-arm)	Retrospective cohort	NA	Single centre	May 2008 to November 2019	Salto Talari’s prosthesis	Patients with primary or post-traumatic ankle DJD who had an ankle fusion using the anterior plate or a TAA.	All groups demonstrate improvement in pain (P<0.001), physical quality of life (P<0.001), and activity (P<0.001) and resulted in a high level of patient satisfaction.	
Lee et al. (2019) [20] (Double-arm)	Retrospective cohort	NA	Single centre	January 2005 to December 2014	HINTEGRA prosthesis	(1) Symptomatic end-stage ankle OA with minimum 36-month follow-up; (2) met TAA indications (good bone stock, normal neurovascular status).	TAA would be a reliable treatment in ankles with ligamentous post-traumatic osteoarthritis when neutrally aligned stable ankles are achieved postoperatively.	
Johnson-Lynn et al. (2018) [17] (Three-arm)	Retrospective cohort	Hospital Joint Registry	Single centre	March 2006 to December 2009	MOBILITY Total Ankle System	Patients who underwent total ankle replacement at the Freeman Hospital.	There was a significant improvement from pre-operatively to 5 years in all scores for patients of 60 years old or younger.	
Bennett et al. (2017) [9] (Five-arm)	Retrospective cohort	Hospital joint registry	Single centre	March 2006 and November 2014	MOBILITY Total Ankle System (DePuy International)	Only patients with 2 years of submitted PROMs were included.	Total ankle replacement may be considered for patients presenting with degenerative ankle arthritis following pilon fracture of the distal tibia, with expectations of similar patient-reported outcomes to patients who undergo primary ankle replacement for osteoarthritis, posttraumatic osteoarthritis, or rheumatoid arthritis.	
Ramaskandhan et al. (2014) [19] (Three-arm)	Retrospective cohort	Hospital Joint Registry	Single canter	March 2006 to December 2009	MOBILITY Total Ankle System (DePuy International)	Patients who underwent TAR with the MOBILITY Total Ankle System.	Early outcomes after total ankle replacement for patients with post-traumatic osteoarthritis are comparable with those for patients with osteoarthritis and rheumatoid arthritis.	
Bai et al. (2010) [21] (Double-arm)	Retrospective cohort	NA	Single centre	January 2005 to July 2007	HINTEGRA prosthesis	Patient with symptomatic ankle arthritis either post-traumatic or primary osteoarthritis.	The incidence of complications after total ankle arthroplasty was higher in the post-traumatic osteoarthritis group than primary osteoarthritis group.	

**Table 4:** Basic characteristics of the double arm studies. Abbreviations: Ankle arthritis (primary) without previous trauma or intervention (POA). PTOA: post-traumatic osteoarthritis; AF: ankle fracture; RA: rheumatoid arthritis; PF: pilon fracture; BMI: body mass index; NA: not available. Sex (male) is reported as number (%). †Variables are reported as mean (SD). *Variables are reported as mean (Range).

Study ID	Study groups		Sample size	Sex (male), N (%)	Age	Mean follow-up (month)	BMI (kg/m²)	
								Megerian et al. (2023) [18]	POA		44	20 (46%)	64 (9)†	39.6	NA	
PTOA		55 (40 malleolar, 14 pilon, 1 talar)	28 (51%)	59 (12)†	37.2	NA		
Albagli et al. (2022) [15]	PTOA		26	11 (42%)	63.8 (10.3)†	32.38	NA	
	Non-traumatic		15	8 (53%)	61.7 (13.4)†	33.24	NA	
Coetzee et al. (2020) [16]	Ankle Fusion for PTOA		100	47 (48.5%)	58.5 (25.2–81.1)*	15.5	34.7 (17.9–61.6)*	
	TAA	POA	100	51 (53.1%)	67.2 (44.9–84.6)*	49.3	30.6 (20.2–49.2)*	
		PTOA	100	56 (57.1%)	64.8 (28.7–94.5)*	76.5	29.0 (16.6–43.5)*	
Lee et al. (2019) [20]	POA		69 ankles (66 patients)	38 (55%)	65.5 (48–78)*	79	25.4 ( 2.73 )†	
	PTOA		50 ankles (48 patients)	28 (56%)	64.1 (38–76)*	71	25.7 (3.57)†	
Johnson-Lynn et al. (2018) [17]	POA		40	31 (77%)	66.7 (47–80)*	60	NA	
	PTOA		27	17 (63%)	58.8 (32–75)*	60	NA	
	RA		9	4 (44%)	63.2 (47–77)*	60	NA	
Bennett et al. (2017) [9]	POA		89	NA	62.4 (11.5)†	24	28.3 (4.3)†	
	PTOA		12	NA	59.4 (19.8)†	24	29.9 (5)†	
	AF		36	NA	64.6 (11.7)†	24	28.4 (4.9)†	
	RA		21	NA	65.1 (10.3)†	24	25.9 (5.1)†	
	PF		15	NA	56.5 (9.7)†	24	31.6 (6.4)†	
Ramaskandhan et al. (2014) [19]	POA		56	41 (73%)	64.4 (10.5)†	24	28.6 (5.35)†	
	PTOA		28	14 (50%)	54.8 (10.8)†	24	30.6 ( 4.21 )†	
	RA		22	10 (45%)	64.2 (10.3)†	24	24.5 (4.23)†	
Bai et al. (2010) [21]	POA		28	26 (70%)	52	38	NA	
	PTOA		37	15 (54%)	64	38	NA	

**Table 5:** Single-arm studies, outcomes of total ankle arthroplasty (TAA) Abbreviations: PTOA: Post-traumatic osteoarthritis; TTA: tibiotalar arthrodesis; NA: not available; PT: physical therapy; CAM: controlled ankle motion; POD: post-operative day; ROM: range of motion; WB: weight bearing; EHL: extensor hallucis longus.

Study ID	Study Design	Number of centres	Study time (recrutement time)	Sample size	Total ankle replacement prosthesis	Etiology (% PTOA)	Inclusion criteria	Main findings (conclusion)	Mean years since TAA	TAA approach	Preoperative medication	Post operative medication		
													Abarquero-Diezhandino et al. (2023) [22]	Retrospective cohort	Two centres	2015 to 2019	50	Trabecular Metal Total Ankle R (Zimmer Biomet)	82%	Patients with end-stage OA, good bone stock, neutral/mild malalignment (<25°), stability, motion preserved.	Transfibular TAA is safe, effective, corrects sagittal/coronal malalignment; excellent clinical/radiological results.	At least 3	Lateral transfibular	NA	Cast until POD 15 → ROM without WB → walker boot → WB at 6 weeks; PT for ROM and stretching		
Fischer et al. (2022) [26]	Retrospective cohort	Single centre	2008 to 2013	148	Tornier Salto (mobile-bearing, n=47) then Salto Talaris (fixed-bearing, n=13)	70%	Patients with end-stage PTOA, age ≥18, informed consent, and treated at study center.	TAA associated with high revision rate after 2nd year; TTA preferred for end-stage PTOA.	NA	Anteromedial	Antiphlogistic meds	Orthotic boot: TTA (12 wks), TAA (8 wks); WB with crutches		
Brodsky et al. (2021) [24]	Prospective cohort	Single centre	NA	33	Scandinavian (n=28) and Salto Talaris (n=5)	30%	Patients with Unilateral primary TAA + preop and 5-year postop 3D gait analysis.	Significant improvement in objective gait parameters compared to preoperative status.	7.6	Anterior (between tibialis anterior and EHL)	NA	PT focused on ROM, strength, gait training. WB in CAM boot at 6 weeks		
Ross et al. 2021 [29]	Retrospective cohort	NA	2010 to 2016	1180	NA	~12%	Preoperative history or active diagnosis of PTOA, traumatic arthropathy, malleolar fractures, etc.	PTOA patients had higher reoperation rates at 1–2 years than non-PTOA TAR patients.	NA	NA	NA	NA		
Rybalko et al. 2018 [30]	Retrospective cohort	NA	1997 to 2010	120	NA	59.20%	NA	TAA use increase 50% in 14 yrs; associated with shorter stays, less rehab, and fewer complications.	NA	NA	NA	NA		
Lee et al. (2013) [25]	Retrospective cohort	2 centres	2003 to 2011	262	Agility (n=197), Salto Talaris (n=56), and STAR (n=9)	46.60%	NA	Radiographic complications common; correlated with worse clinical outcomes.	NA	NA	NA	NA		
Rodrigues-Pinto et al. (2013) [28]	Prospective cohort	18 centres	2005 to 2011	119	Salto1	65.50%	NA	Proper patient selection + surgical technique reduces complications and improves survival.	39	Anterior incision between tibialis anterior & EHL tendons	Prophylactic antibiotics	Antibiotics 24h, antithrombotics for 4 weeks		
Noelle et al. (2013) [27]	Retrospective cohort	Single centre	2005 to 2010	97	S.T.A.R.	81%	NA	High satisfaction and pain relief; BMI >30 linked to more complications.	36	Anterolateral midline incision	NA	Cephalosporin 24h; immobilization 48–72h		
Bardelli and Scoccianti (2006) [23]	Retrospective cohort	NA	1997 to 2004	7	S.T.A.R. uncemented TAA	100%	Patients with PTOA post fracture, ≥3 years FU, available for clinical/radiographic re-eval.	TAA in PTOA is technically difficult; outcomes should be evaluated separately from POA or RA cases.	5.1	NA	Cephalosporin + Heparin	Below-knee cast x30 days, immediate WB; physiotherapy after cast removal		

**Table 6:** Basic characteristics of the single-arm studies Abbreviations: TAA: Total ankle arthroplasty; BMI: body mass index; NA: not available. Sex (male) is reported as % (number/total), Other dichotomous variables are reported as number (%). † Variables are reported as mean (SD). *Variables are reported as mean (range).

Study ID	Group	Age	Sex (male), % (N)	BMI (kg/m2)	Diabetes N (%)	Hypertension N (%)	Mean follow-up (months)
Abarquero-Diezhandino et al. (2023) [22]	TAA	59 (39 - 81)*	NA	NA	NA	NA	45
Fischer et al. (2022) [26]	TAA	57.05 ( 38 - 77)*	58.3% (35/60)	29.42 ( 21.39 - 43.44)*	5 (8.3)	NA	NA
Brodsky et al. (2021) [24]	TAA	61.1 (11.8)†	24% (8/33)	28 (4.9)†	2 (6)	NA	7.6
Ross et al. (2021) [29]	TAR	NA	47.7 %(151/318)	NA	108 (34.0)	219 (68.9)	NA
Rybalko et al. (2018) [30]	TAA	57.8 (19–83)*	42.5% (51/120)	NA	8 (6.7%)	NA	NA
Lee et al. (2013) [25]	TAA	61.5 (NA)†	45% (117/260)	NA	NA	NA	35.5
Rodrigues-Pinto et al. (2013) [28]	TAA	55.6 (24–81)*	55.5% (66/119)	NA	NA	NA	38.7
Noelle et al. (2013) [27]	TAA	63 (41–80)*	45% (33/97)	28.4 (21.7–38.6)*	NA	NA	36
Bardelli and Scoccianti (2006) [23]	TAA	59.1 (30–76)*	43% (3/7)	NA	NA	NA	60.8

Risk of Bias Assessment

Regarding the NIH Quality Assessment Tool of single-arm retrospective cohort studies, two studies demonstrated an overall good quality [24,28], five studies demonstrated an overall fair quality [22,25-27,30], and two studies demonstrated an overall poor quality [23,29]. Detailed NIH domains are shown in Table 2. Regarding Newcastle Ottawa Scale (NOS), five studies demonstrated an overall good quality [9,15,18,20,21], and three studies demonstrated an overall fair quality [16,17,19]. Detailed NOS domains were shown in Table 1.

Revision Rate

**Figure 2:**
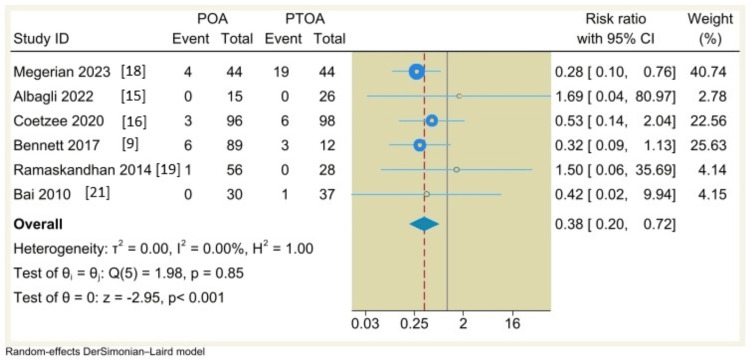
Forest plot comparing revision rates between non-traumatic arthritis (POA) versus post-traumatic osteoarthritis (PTOA) following total ankle arthroplasty.

Six included studies reported the revision rate with follow-up duration from 0.1 to 11.2 years with an incidence rate of 4.2% (14 of 330 patients) in the POA group compared to 11.8% (29 of 245 patients) in the PTOA group. The pooled analysis using a random-effects DerSimonian-Laird model indicated a significant difference between POA and PTOA groups (RR=0.38, 95% CI: 0.2 to 0.72), P<0.001; I^2^= 0%, p=0.85), as shown in Figure 2.

AOFAS Ankle-Hindfoot Score

**Figure 3:**
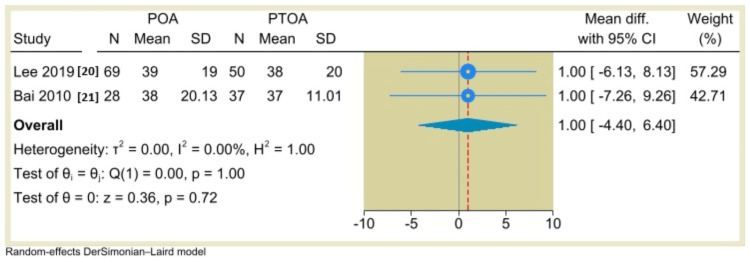
Forest plot comparing postoperative AOFAS scores between non-traumatic arthritis (POA) and post-traumatic osteoarthritis (PTOA) after total ankle arthroplasty.

**Figure 4:**
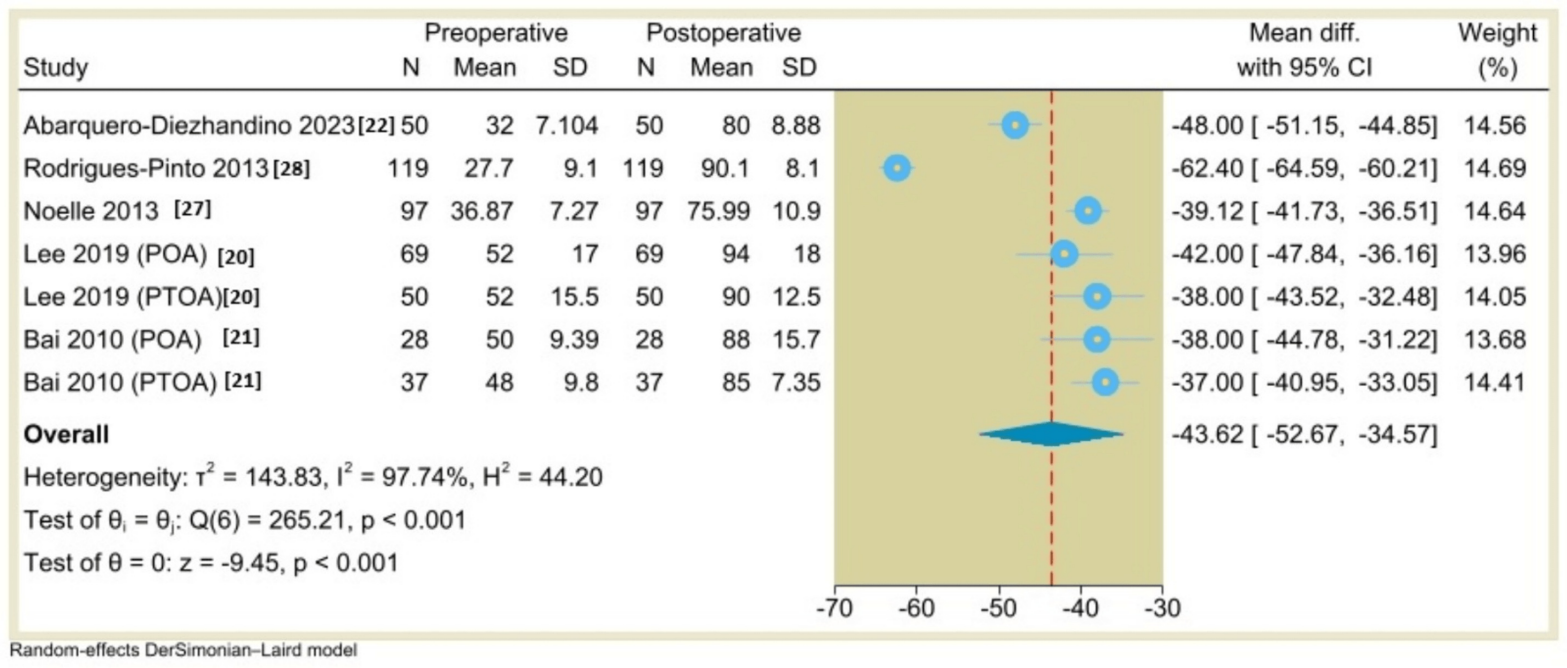
Forest plot of mean change in AOFAS scores from preoperative to postoperative period following total ankle arthroplasty.

The AOFAS score a reported outcome in five studies. However, only two studies reported extractable means and dispersion for the analysis. A random-effects DerSimonian-Laird model was used to compare the effect of TAA in both POA and PTOA groups. The pooled analysis indicated a statistically nonsignificant difference between POA and PTOA groups (MD=1.0, 95% CI: -4.4 to 6.4, P=0.72) (Figure 3). The single-arm meta-analysis of five studies (seven groups) showed that the AOFAS score was -43.62, 95% CI: -52.67 to -34.57, P<0.001 (Figure 4).

Complication Rates

**Figure 5:**
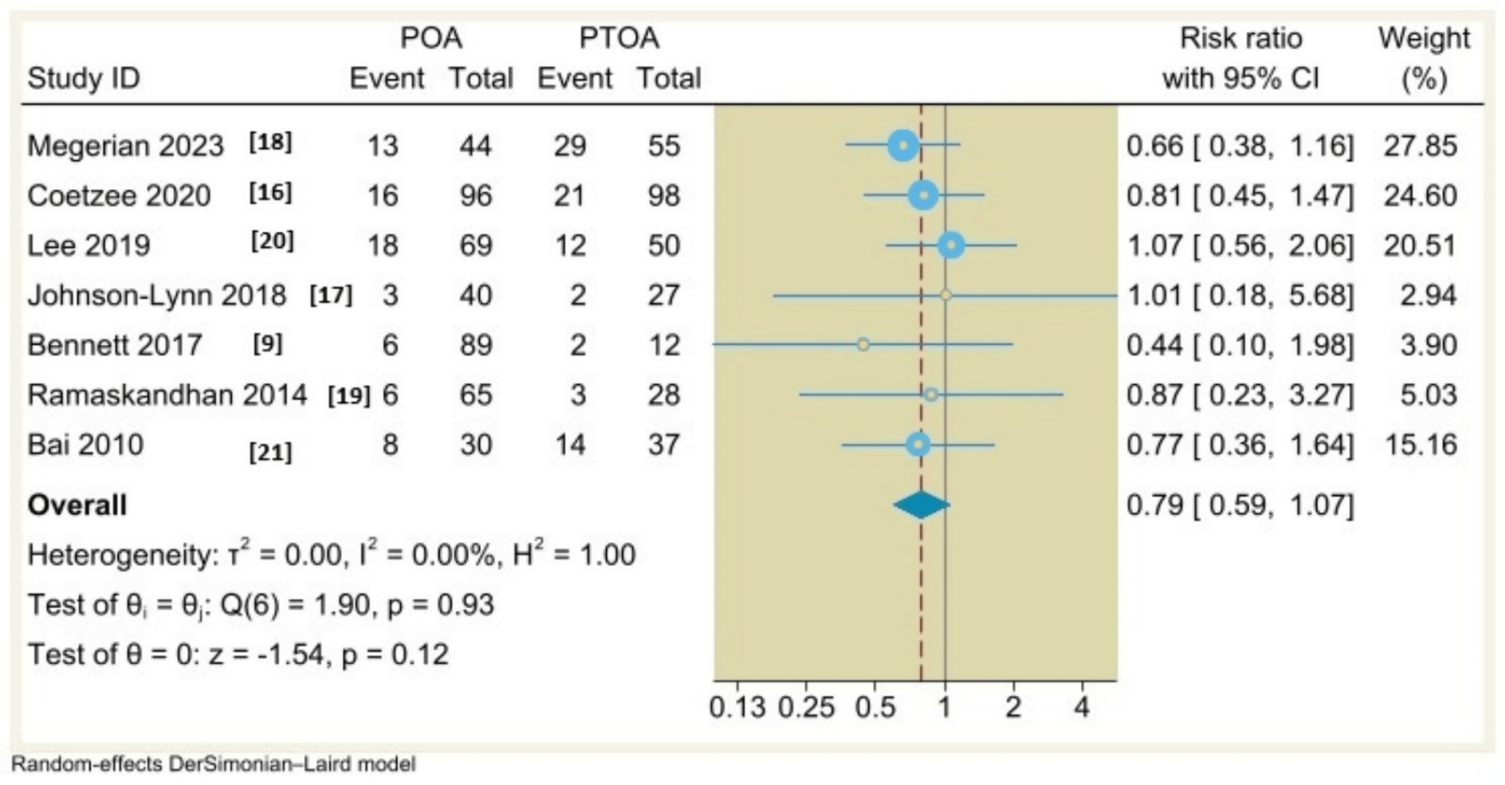
Forest plot comparing overall complication rates between non-traumatic (POA) and post-traumatic osteoarthritis (PTOA) following total ankle arthroplasty

**Figure 6:**
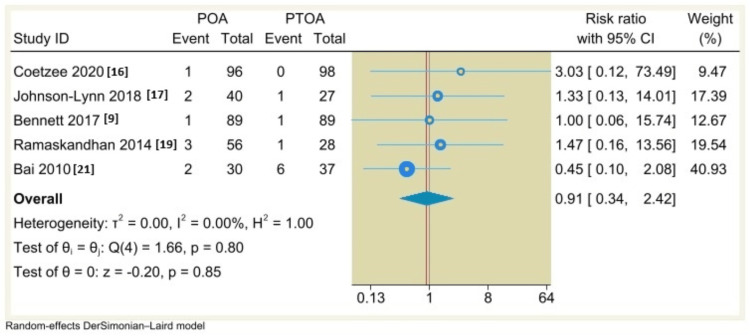
Forest plot comparing malleolar fracture rates between non-traumatic arthritis (POA) and post-traumatic osteoarthritis (PTOA) following total ankle arthroplasty.

Regarding complication rate and malleolar fracture, we polled the results from seven and five included studies, respectively. By using a random-effects DerSimonian-Laird model, there is no significant difference between POA and PTOA (RR=0.79, 95% CI: 0.59 to 1.07, P=0.12; I^2^= 0%, p=0.93) (Figure 5); (RR=0.91, 95% CI: 0.34 to 2.42, P=0.85; I^2^= 0%, p=0.8) (Figure 6).

Single-arm Meta-Analysis

**Figure 7:**
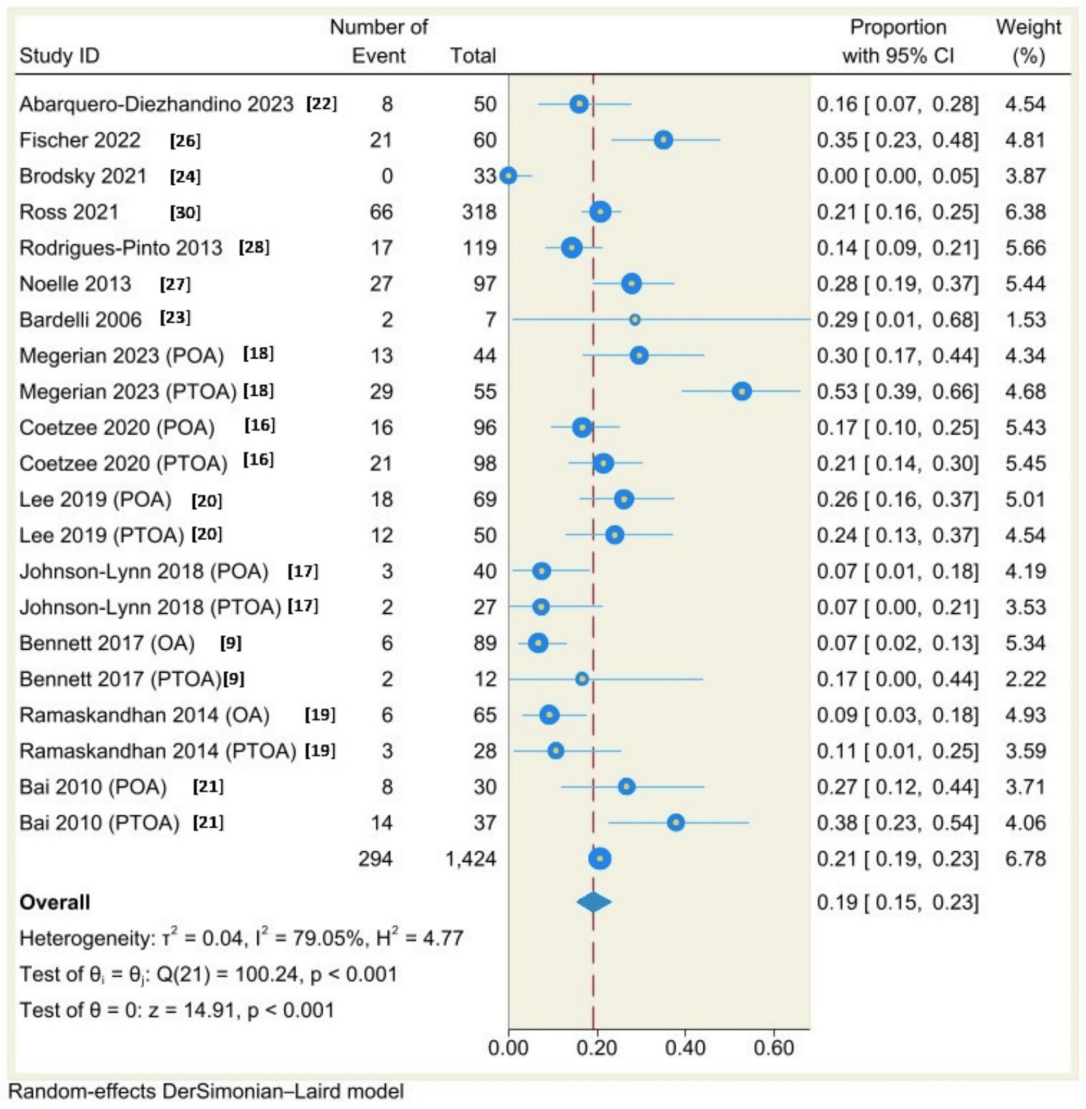
Forest plot of overall complication rates following total ankle arthroplasty.

**Figure 8:**
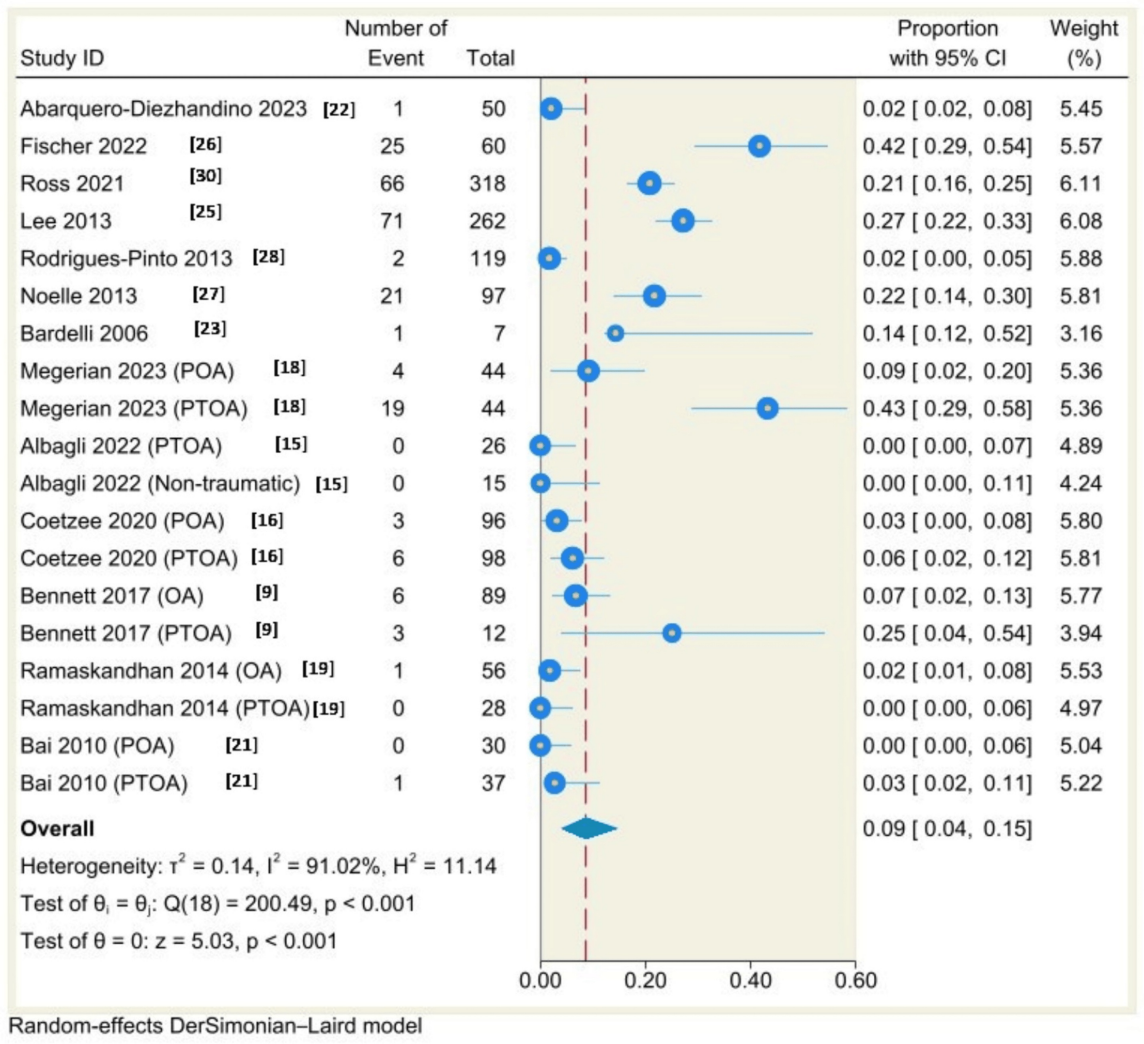
Forest plot of revision rates following total ankle arthroplasty.

**Figure 9:**
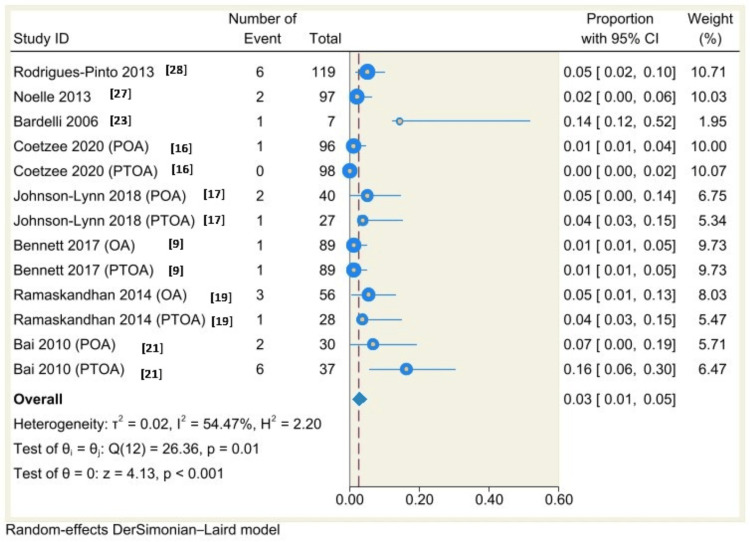
Forest plot of malleolar fracture rates following total ankle arthroplasty.

Using a random-effects model (DerSimonian-Laird) and Freeman-Tukey double arcsine transformation, the results of single-arm meta-analysis of 14 studies (21 groups) showed that the complication rate was 19% (95% CI: 15% to 23%, P<0.001) with considerable heterogeneity (I^2^=79.05%, P<0.001) (Figure 7), the results of single-arm meta-analysis of 13 studies (19 groups) showed that the revision rate was 9%, 95% CI: 4% to 15%, P<0.001 with considerable heterogeneity (I^2^=91.02%, P<0.00) (Figure 8), the results of single-arm meta-analysis of eight studies (13 groups) showed that the incidence of malleolar fracture was 3%, 95% CI: 1% to 5%, P<0.001 with moderate heterogeneity (I^2^=54.47%, P=0.01) (Figure 9).

**Figure 10:**
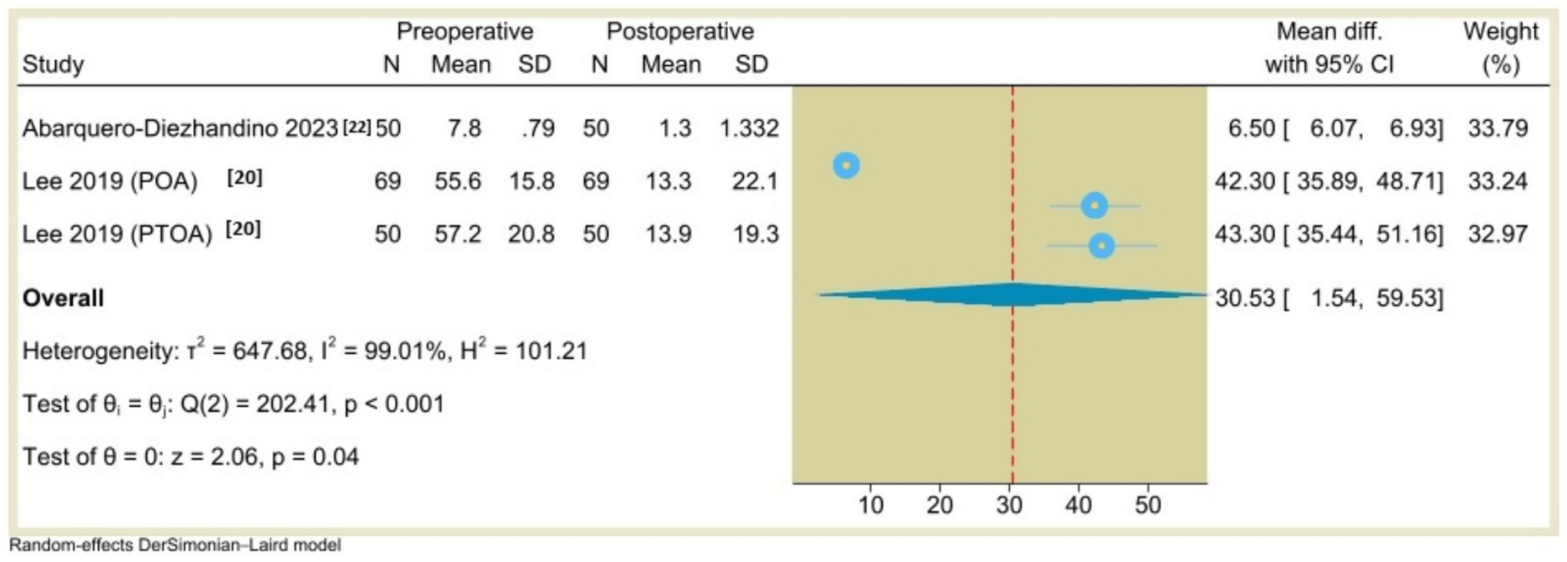
Forest plot of mean change in pain scores (Ankle Osteoarthritis Scale (AOS)) from preoperative to postoperative period following total ankle arthroplasty.

**Figure 11:**
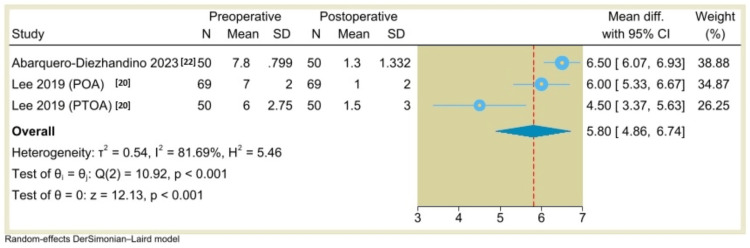
Forest plot of mean change in Visual Analogue Scale (VAS) pain scores from preoperative to postoperative period following total ankle arthroplasty.

**Figure 12:**
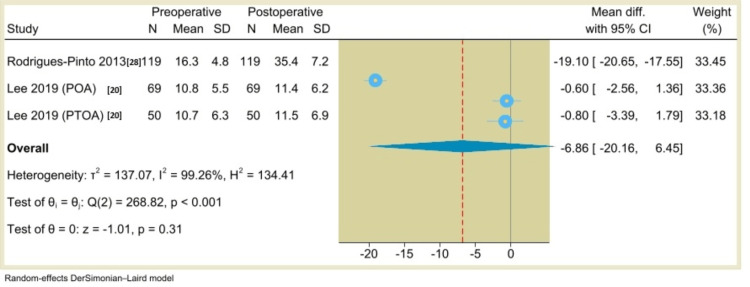
Forest plot of mean change in ankle dorsiflexion range of motion from preoperative to postoperative period following total ankle arthroplasty.

**Figure 13:**
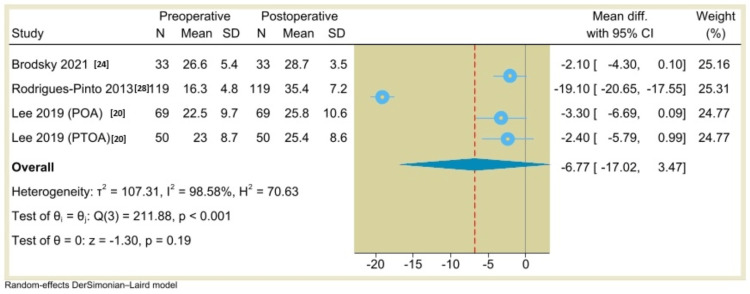
Forest plot of mean change in ankle plantarflexion range of motion from preoperative to postoperative period following total ankle arthroplasty.

Additionally, the results of single-arm meta-analysis showed that the AOS (pain) score was 30.53 (95% CI: 1.54 to 59.53, P=0.04) (Figure 10), the VAS score was 5.8 (95% CI: 4.86 to 6.74, P<0.001) (Figure 11), the ROM (dorsiflexion) was -6.86 (95% CI: -20.16 to 6.45, P=0.31) (Figure 12), and the ROM (plantarflexion) was -6.77 (95% CI: -17.02 to 3.47, P=0.19) (Figure 13) with considerable heterogeneity (I^2^=99.01%, P<0.001), (I^2^=81.69%, P<0.001), (I^2^=99.26%, P<0.001), and (I^2^=98.58%, P<0.001), respectively.

Discussion

To our knowledge, this is the first meta-analysis comparing outcomes of TAA in patients with POA and PTOA. The meta-analysis of the included studies found no statistically significant difference in the primary outcome, measured as the mean difference in AOFAS scores pre- and post-operatively between the two groups [20,21]. The AOFAS scores improved significantly from baseline in both groups, indicating favourable short-term functional outcomes. However, the meta-analysis revealed a statistically significantly higher revision rate for TAA in the PTOA group compared to the POA group. Our findings suggest a role for TAA in patients with post-traumatic osteoarthritis, though its application may need to be individualised based on patients’ functional demands, comorbidities, expectations and perceived surgical complexity.

These findings are similar to another systematic review, which showed significant improvement in the functional outcomes after total ankle arthroplasty, where the AOFAS score changed from 40 (95% CI: 36 to 43) pre-operatively to 80 (95% CI: 76 to 84) at a mean follow-up of 8.2 years (7-10) (p<0.01) [31]. Our meta-analysis showed a slight reduction in the ROM after TAA, but the reduction was not statistically significant. In comparison, another study of three-dimensional gait analysis of TAA reported a seven-year significant improvement in gait parameters, such as the step length and walking speed and range of motion. There were increases in total sagittal range of motion (+2.0°; p=0.0263), plantar flexion at initial contact (+2.7°; p=0.0044), and maximum plantar flexion (+2.0°; p=0.0488) [24].

Data from the 2024 National Joint Registry (NJR) report estimated the five-year post operative revision rate for primary TAA to be from 2.52% (95% CI 1.96-3.23) to 8.47% (95% CI 6.97-10.28), with the most common cause being aseptic loosening followed by infection; 0.43 per 100 prosthesis-years 95% CI (0.38-0.49) and 0.29 per 100 prosthesis-years (95% CI: 0.25-0.34) [32]. In this review, the identified risk factors for revision in patients with PTOA were preoperative valgus malalignment [18], periprosthetic osteolysis and loosening, technical errors, deep infections [9,20,21], varus instability, talar subsidence due to avascular necrosis [17 ,9 ], medial subluxation and polyethylene dislocation [19].

Our meta-analysis found no statistically significant difference in complication rates between the POA and PTOA groups, with low statistical heterogeneity among studies (I²=0%). These findings should be interpreted cautiously due to small sample sizes; larger randomised controlled trials are needed to confirm these results. In another systematic review [33], the most common reported complication was an intraoperative malleolar fracture of 6.0 % (95% CI: 4.0-8.0%; Grading of Recommendations Assessment, Development and Evaluation (GRADE) very low). In our meta-analysis, the incidence of intraoperative malleolar fracture was 3% (95% CI: 1% to 5%, P<0.001).

Limitations

There is paucity of high-level, long-term longitudinal studies that aim to ascertain the differences in outcomes between TAA in POA and PTA. This meta-analysis showed no statistically significant difference in primary outcomes in the short term; however, this result was drawn from only two studies. It is, however, reassuring that the subgroup meta-analysis of intra-operative complications such as malleolar fractures, which was derived from a wider evidence base, was also not statistically significant.

Some authors argue that POA may not be a distinct clinicopathological entity, suggesting that cases classified as POA could represent PTOA with unrecognised prior trauma, which may have been subclinical. Consequently, this meta-analysis could be interpreted as comparing outcomes of TAA in unrecognised PTOA without surgical intervention versus PTOA with surgical intervention. However, this perspective does not negate the surgical or anatomical differences between the two cohorts, nor does it undermine the observed complications, perceived surgical concerns, or reported outcomes. Accordingly, clinicians should either apply the current evidence base to clinical practice or conduct further studies to address these valid concerns.

The primary outcome was assessed using the AOFAS scale. The validity of the meta-analysis therefore depends on the scale’s validity, reliability, and responsiveness. Available data on ankle osteoarthritis indicates that the patient-reported component of the AOFAS score is valid and responsive [34]. However, the minimally important change (MIC) for the AOFAS score in ankle OA has not been established, limiting insight into what constitutes a clinically significant change. For example, a patient’s AOFAS score may increase from 65 to 70 between follow-ups, but this difference may not reflect meaningful functional improvement [35]. Furthermore, the generalisability and applicability of the AOFAS score may be constrained by potential floor and ceiling effects.

## Conclusions

This meta-analysis demonstrates that total ankle arthroplasty in patients with prior surgical intervention for post-traumatic arthritis is associated with higher revision rates than in patients without previous intervention. However, they might still yield similar functional outcomes in the short term. Advancements in implant design and surgical techniques have broadened the indications for TAA. TAA is effective for patients with PTOA, whether or not they had prior surgical intervention. Short-term complication rates may have been historically overestimated, but long-term risks remain a concern. The role of TAA should be clearly defined, weighing risks against patient expectations, with longitudinal studies needed to further clarify such risks and benefits.
